# Functional In Vitro Expression of the Succinate–Acetate Transporter SatP and an Initial Screening Strategy

**DOI:** 10.3390/membranes16080252

**Published:** 2026-07-23

**Authors:** Yuelang Yao, Bernhard Schuster, Karola Vorauer-Uhl, Diethard Mattanovich

**Affiliations:** 1 Institute of Microbiology and Microbial Biotechnology, Department of Biotechnology and Food Science, BOKU University, 1190 Vienna, Austria; 2 Institute of Bioprocess Science and Engineering, Department of Biotechnology and Food Science, BOKU University, 1190 Vienna, Austria; 3 Institute of Synthetic Bioarchitectures, Department of Biotechnology and Food Science, BOKU University, 1190 Vienna, Austria

**Keywords:** SatP, in vitro protein expression, cell-free expression, acetate–succinate transporter, single-channel recording

## Abstract

In vitro protein expression has gained considerable attention due to its experimental flexibility and time efficiency. This method employs essential transcriptional components and is particularly advantageous for expressing proteins that are challenging to produce in living systems, such as membrane or toxic proteins. Membrane acetate transporters regulate cellular acetate levels and are important for microbial engineering in industrial fermentation processes. In this study, the succinate–acetate transporter protein SatP from *Escherichia coli* was functionally expressed utilizing a cell-free system and subsequently characterized through single-channel recordings. Beyond confirming its transport activities, we identified malate and fumarate as additional potential substrates of SatP. The successful in vitro expression of SatP lays the foundation for cell-free screening of acetate transporters. We then further investigated a preliminary screening assay and proposed a potential strategy for the rapid expression and functional characterization of acetate transport proteins. Because acetate accumulation can inhibit microbial growth and reduce fermentation efficiency, a better understanding of SatP-mediated acetate transport may facilitate the engineering of microbial strains with enhanced acetate tolerance and improved product yields. This knowledge provides a foundation for the rational optimization of industrial fermentation processes.

## 1. Introduction

In vitro protein expression, also termed cell-free expression, employs an open reaction environment that contains the essential transcription–translation machinery without living cells. It is typically conducted in microliter-scale reactions initiated by adding plasmid templates, resulting in protein production within a few hours.

This boundary-free setup is particularly advantageous for the expression of toxic proteins, whose overproduction in living cells often causes cellular stress or even cell death, resulting in poor protein yields [1,2]. Membrane proteins are a notable example, as their overexpression can compromise membrane integrity, saturate the translocation machinery, and promote protein misfolding and aggregation. Cell-free expression systems can circumvent these limitations by enabling protein synthesis in a controlled environment without the constraints of maintaining cellular viability, often leading to improved expression and solubility of the membrane proteins [3].

Owing to their open and adaptable reaction environment, cell-free expression systems have also been developed into platforms for rapid protein characterization and functional screening. Functional screening of soluble proteins using cell-free expression systems has been widely explored, including applications involving antibody fragments [4], AMPylated proteins [5], and mutational variants [6]. Although the optimization of expression and expression-based screening in membrane proteins have been thoroughly investigated [7,8,9], direct in situ functional screening following membrane protein expression remains relatively uncommon [10].

Acetate, a major by-product of microbial fermentation, inhibits cell growth and productivity at elevated concentrations. Engineering acetate transporters to enhance activity and specificity can improve microbial acetate tolerance, making a mechanistic understanding of these transporters essential for optimizing industrial fermentation processes. The Acetate Uptake Transporter (AceTr) family comprises transmembrane proteins that mediate acetate import and export. Transporters from the AceTr family were characterized by acetate transporting functions including SatP from *Escherichia coli* [11], Ady2 from *Saccharomyces cerevisiae* [12], Gpr1 from *Yarrowia lipolytica* [13] and AcpA from *Aspergillus nidulans* [14]. Among the members of the AceTr family, SatP is one of the most well-characterized transporters. It was first identified as a succinate–acetate transporter by Sá-Pessoa et al. [11]. More recently, the crystal structure of SatP from *E. coli* revealed that SatP forms hexamers composed of identical monomers [15]. The ortholog of *E. coli* SatP from *Citrobacter koseri* has a similar hexameric structure and shares acetate and succinate transport capabilities with *E. coli* SatP [16]. The molecular mechanism of acetate transport by SatP has been further characterized, showing that acetate is transported through the channel of a single monomer, with the narrowest constriction located at its center [15,16]. The potential of engineered acetate transporters for higher biofuel production has also been explored. Functional expression of SatP in the yeast fermentation strain *S. cerevisiae* demonstrated that the L131A mutation increased lactate and succinate import as well as acetate export in vivo. These findings also highlight the potential of engineered acetate transporters in metabolic engineering strategies that aim at optimizing industrial fermentation and biofuel production.

Here, we explored the feasibility of expressing the succinate–acetate transporter SatP in a cell-free expression system. Following in vitro synthesis, the transport activity of SatP toward different substrates was analyzed by the single-channel recording technique [17,18,19]. In addition, we established a transporter screening strategy that serves as the basis for a rapid and efficient platform for transporter activity assessment. Because acetate accumulation inhibits microbial growth during fermentation, the screening strategy presented here provides a foundation for the rapid characterization and engineering of microbial strains with enhanced acetate tolerance. With further development, this approach holds promise for the optimization of fermentative microorganisms and a broad range of biotechnological applications.

## 2. Materials and Methods

### 2.1. Plasmid Construction

**Figure A1:**
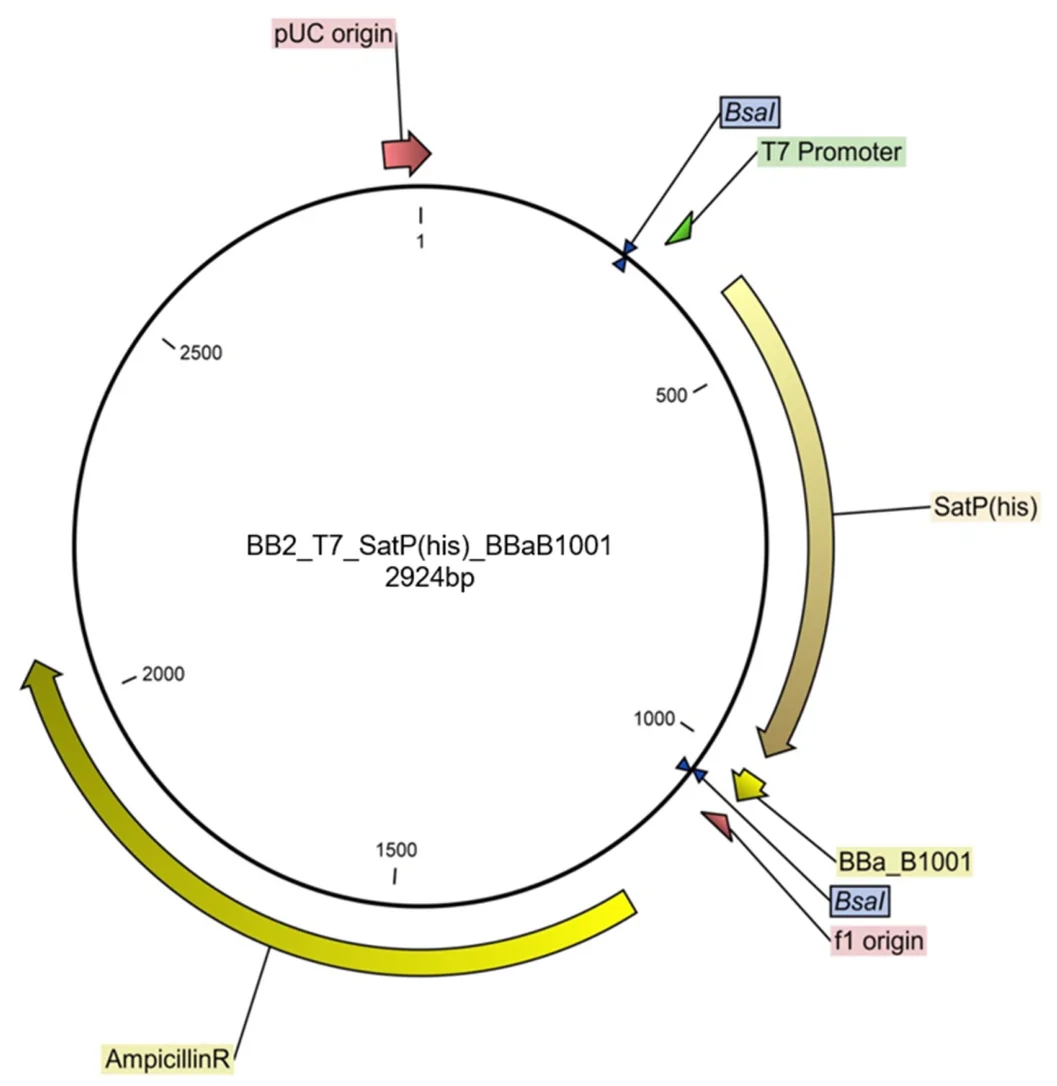
Plasmid information of BB2_T7_SatP(his)_BBaB1001. The plasmid contains a T7 promoter, a His-tagged SatP gene, and a BBa_B1001 terminator. Additional features include an ampicillin resistance gene, BsaI restriction sites, a pUC origin of replication, and an f1 origin of replication.

The plasmids were constructed using Golden Gate assembly, and the methods were adapted from previous protocols [20,21]. Plasmids containing T7 promoter, SatP with 6 histidine tags at C terminus, and BBa_B1001 terminator were constructed on backbone 2, named BB2_T7_SatP(his)_BBaB1001. In the first step, 3 plasmids, backbone 1 containing T7 promoter, backbone 1 containing SatP(his), and backbone 1 containing BBa_B1001 were constructed independently. Then 3 plasmids were digested using type II restriction enzymes and assembled to construct BB2_T7_SatP(his)_BBaB1001 plasmids (Figure A1).

### 2.2. SatP Synthesis in Cell-Free Expression

The RTS 100 *E. coli* HY Kit (biotechrabbit GmbH, Berlin, Germany) was used for cell-free expression; the reaction was incubated at 30 °C for 3 h and was stopped by being transferred onto ice. In a 50 μL reaction, 12 μL *E. coli* lysate, 510 μL reaction mix, 12 μL amino acids, 1 μL methionine 5 μL reconstitution buffer, 800 ng plasmids, and 3 μL 20 mg/mL liposomes were added according to the instructional protocol.

### 2.3. Liposome Preparation

Liposomes were generated using a film rehydration approach. A total of 50 mg of *E. coli* polar lipid extract (Avanti Polar Lipids, Darmstadt, Germany) was dissolved in 2.5 mL chloroform. The solvent was removed with a rotary evaporator (BÜCHI, Flawil, Switzerland) at 40 °C, applying 300 mbar for 10 min followed by 10 mbar for 15 min, resulting in a uniform lipid film on the flask wall. The dried lipids were resuspended in 2.5 mL buffer (450 mM NaCl, 5 mM bis-tris propane, pH 7.5) and rotated at 40 °C for 1 h. The resulting suspension was extruded sequentially through membranes of 400 nm, 200 nm, and 100 nm channel size (Whatman, GE Healthcare, Maidstone, Kent, UK) using a LIPEX^®^ extruder (Northern Lipids Inc., Burnaby, Vancouver, BC, Canada); there were seven cycles per step at pressures up to 25 mbar, with the device maintained at 40 °C. In each batch of liposomes, the particle size distribution was measured by dynamic light scattering (Zetasizer Nano ZS, Malvern Panalytical Ltd., Malvern, Worcestershire, UK) 3 times, and the average size distribution was recorded.

### 2.4. Plate-Based SatP Expression

In the plate-based screening assay, liposomes were formulated with an additional 10 µL of Biotin-X-DHPE (10 mg/mL in chloroform). Streptavidin-coated high-capacity plates (Pierce™, Thermo Fisher Scientific, Rockford, IL, USA) were used to immobilize liposomes. Biotinylated liposomes were added to each well and incubated for 30 min at room temperature with orbital shaking at 180 rpm. Unbound liposomes were removed, and the wells were washed three times with 200 µL buffer (450 mM NaCl, 5 mM bis-tris propane, pH 7.5), each wash performed with shaking at 180 rpm for 5 min.

For SatP synthesis, 50 µL of the cell-free expression mixture was added to each well after the final wash, and plates were incubated for 3 h at 30 °C with shaking at 180 rpm for SatP expression. Following expression, the reaction mixture was removed, and the wells were again washed three times with the same buffer under identical conditions. Negative controls were prepared in parallel using expression mixtures without plasmid DNA, while all other procedures were identical.

For Western blot, 15 µL 1% n-dodecyl-β-D-maltoside (DDM) was added after the last washing buffer was aspirated to solubilize proteoliposomes for further analysis. For peptide digestion, 1 µL proteinase K (NEB #P811 Thermolabile Proteinase K) in 100 µL, 50 mM, pH 7.0 PBS buffer was added to each well after the last washing buffer was aspirated and was incubated at 37 °C for 40 min. The resulting supernatant was subjected to mass spectrometry.

### 2.5. Mass Spectrometry

Mass spectrometry was performed by BOKU core facility. After the samples were digested by protease K, they were loaded on a nanoEase C18 column (nanoEase M/Z HSS T3 Column, 100 Å, 1.8 μm, 300 μm × 150 mm, (Waters GmbH, Vienna, Austria) using 0.1% formic acid as the aqueous solvent. A gradient from 1% B (B: 80% Acetonitrile, 0.1% FA) to 40% B in 50 min was applied, followed by a 10 min gradient from 40% B to 95% B that facilitates elution of large peptides, at a flow rate of 6 μL/min.

Mass spectrometric analyses were performed on an Orbitrap Exploris 480 mass spectrometer (Thermo Fisher Scientific, Rockford, IL, USA) equipped with a heated electrospray ionization (H-ESI) source operated in positive ion mode. The spray voltage was set to 3.4 kV, the ion transfer tube temperature to 320 °C, and the auxiliary gas flow to 7 (arbitrary units), while the sheath and sweep gas flows were set to zero. The total duration of the method was 30 min. Internal mass calibration was performed using a user-defined lock mass at *m*/*z* 445.12003 with a mass tolerance of 15 ppm.

Data acquisition was carried out in data-dependent acquisition (DDA) mode with a cycle time of 2 s. Full MS scans (MS1) were acquired in the Orbitrap at a resolution of 60,000 (at *m/z* 200) over a mass range of *m/z* 375–1200. The maximum injection time was set to 25 ms. MS1 data were acquired in profile mode.

Precursor ion selection was restricted to peptides with charge states of +1 to +6 and a minimum intensity threshold of 8000. Dynamic exclusion was enabled with a repeat count of 1 and an exclusion duration of 20 s, using a mass tolerance of ±10 ppm. Isotopic ions were excluded, and only one charge state per precursor was selected for fragmentation.

For MS/MS acquisition, precursor ions were isolated using an isolation window of 1.4 *m/z* and fragmented by higher-energy collisional dissociation (HCD) with normalized collision energies of 28%, 30%, and 32%. Fragment ions were detected in the Orbitrap at a resolution of 15,000. The maximum injection time was 75 ms with an AGC target of 100% (custom setting). MS/MS spectra were acquired in centroid mode with a lower mass limit of *m/z* 120.

Instrument calibration was performed using Pierce FlexMix Calibration Solution (Thermo Fisher Scientific, Rockford, IL, USA). Data was processed using PEAKS X Pro Studio 10.6, which is suitable for performing MS/MS ion searches. The analysis was searched against the *E. coli* database or the target protein.

### 2.6. Single-Channel Recording Using Planar Bilayer

Single-channel recording of SatP was conducted following a previously established protocol [22]. A bilayer composed of 1,2-diphytanoyl-*sn*-glycero-3-phosphocholine (DPhPC) was formed on an aperture measuring 150 µm in diameter within a Teflon septum, which was 25 µm thick and served to divide the custom-made chamber into *cis* and *trans* compartments (4 mL each). Single-channel recordings were performed using a patch-clamp amplifier (HEKA EPC10 USB, Lambrecht, Germany) with PatchMaster software (version 2 × 92) and a two-electrode setup using AgCl-coated silver wires as reference and recording electrodes. The setup was placed on a vibrationally isolated table within a grounded Faraday cage. All measurements were carried out in electrolyte solutions (see Table A1) at 22 °C. The holding potential was ±150 mV; measurements were taken for 10 s. The settings of the two built-in band filters were 10 and 2.9 kHz, respectively.

**Figure 1:**
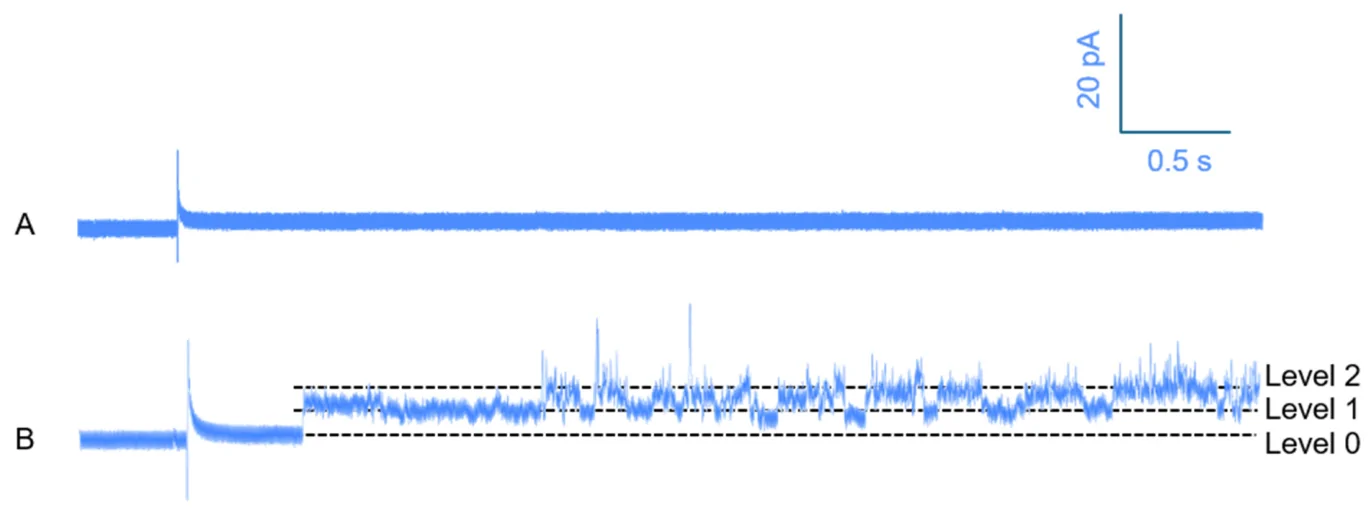
Single-channel recording of SatP in acetate buffer. (**A**) Current versus time trace of the lipid bilayer in the absence of SatP. The spike observed after 0.5 s signifies the application of the voltage at +80 mV. (**B**) Current versus time trace with SatP integrated into the lipid bilayer upon applying +80 mV voltage. Dashed lines denote the current levels; the lowest one (level 0) corresponds to the membrane without the incorporation of a functional SatP channel. When one SatP is functionally reconstituted, a stepwise increase in current is observed (level 1). A second opening of the SatP channel gives rise to another stepwise increase in current (one step) and brings the current to level 2.

To measure the substrate transport, a 10 mM HEPES buffer at pH 7.0 comprising 200 mM substrate was transfered into the cis- and trans-compartments (Table A1). Blank measurements were conducted without the presence of SatP under varying voltages. Subsequently, 5 μL of SatP–proteoliposomes, synthesized via cell-free expression, was introduced into both compartments. The incorporation of SatP was confirmed by stepwise changes in current under specific voltages. In a buffer containing the SatP substrate, but lacking SatP incorporation into the lipid bilayer, the application of a voltage did not induce any current change (Figure 1A). Conversely, whenever SatP was incorporated into lipid bilayers, current changes were observed (Figure 1B). From these measurements, the smallest abrupt current change (one step) was identified as a single transport event by an individual SatP (Figure 1B). The steps observed at different applied voltages were plotted to generate an I–V curve. When the I–V curve exhibited a linear relationship, the slope value was determined through linear regression, representing the single-channel conductance of SatP. A 10 mM HEPES buffer at pH 7.0 comprising 200 mM substrate was used to measure transport of other substrates and NaCl (Table A1).

### 2.7. Protein Electrophoresis

SDS-PAGE was run using 10% NuPAGE™ Bis-Tris Protein from Thermo Fisher Scientific, Rockford, IL, USA and run according to the instructional manuals.

Western blot was conducted using the Trans-Blot Turbo Transfer System (Bio-Rad Laboratories GmbH, Vienna, Austria) as instructed. The Trans-Blot Turbo Mini 0.2 µm PVDF Transfer Pack was used for transfer. Following the transfer, the membrane was blocked in 5% skimmed milk and 0.05% PBST with gentle shaking for 1 h, and then was washed with 0.05% PBST. The membrane was incubated for 1 h in 10 mL 5% skimmed milk and 0.05% PBST with 1 µL of anti-polyhistidine–peroxidase antibody (Sigma-Aldrich Handels GmbH, Vienna, Austria) and was washed again. Subsequently, the HRP chemiluminescent substrate (SuperSignal West Pico Chemiluminescent Substrate, Thermo Fisher Scientific, Rockford, IL, USA) was carefully poured over the membrane for chemiluminescent imaging.

### 2.8. Computational Structural Analysis

The structure of SatP was visualized using PyMOL (version 2.5.2, Schrödinger, LLC, New York, NY, USA). Structural coordinates were obtained from the Protein Data Bank (PDB ID: 5ZUG). Both hexameric and monomeric assemblies were visualized. The default hexameric assembly was used and displayed in the cartoon representation. Then the structure was rotated by 90°, with the periplasmic side at the top and the cytoplasmic side at the bottom. A monomer SatP was extracted from the hexamer and visualized in the same orientation, the periplasmic side at the top and the cytoplasmic side at the bottom.

## 3. Results

### 3.1. SatP Synthesized in Cell-Free Expression

**Figure 2:**
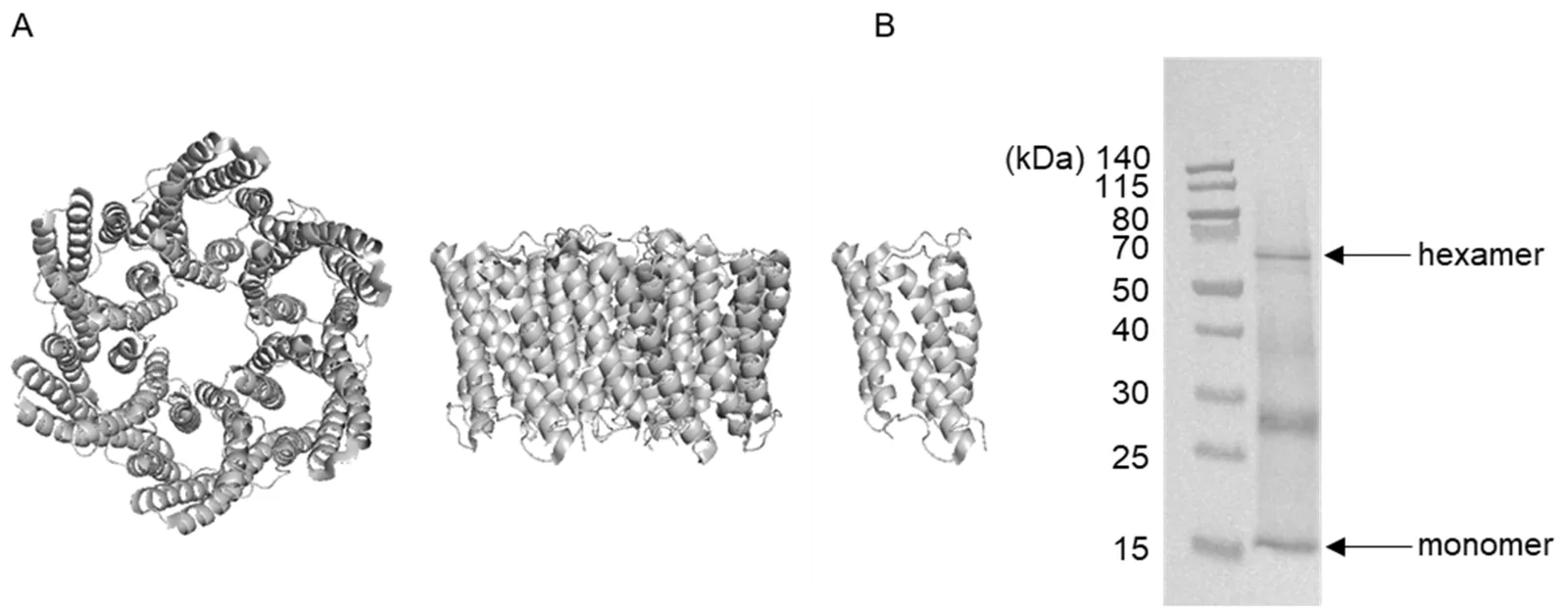
Cell-free expression of *E. coli* SatP. (**A**) SatP (PDB ID: 5ZUG) structures were visualized using PyMOL 2.5.2. Hexameric SatP is shown in the default PyMOL orientation (left), the periplasmic side at the top and the cytoplasmic side at the bottom (middle), and a monomeric SatP with the periplasmic side at the top and the cytoplasmic side at the bottom (right). (**B**) Western blot analysis of SDS-PAGE. His-tagged SatP was expressed using a cell-free expression system in the presence of liposomes. Following expression, the reaction mixture was subjected to SDS-PAGE and analyzed by Western blotting. Bands corresponding to monomeric and hexameric forms of SatP are indicated alongside the blot.

*E. coli* SatP, a membrane succinate–acetate transporter that assembles into a hexamer of six identical monomers (Figure 2A), was synthesized using a cell-free expression system in the presence of liposomes.

The expression was analyzed by Western blot following SDS-PAGE (Figure 2B). Despite SDS treatment, SatP was not fully dissociated into its monomeric form, as multiple immunoreactive bands were observed in the Western blot. Similar observations have previously been reported in SatP from both *E. coli* and *C. koseri* [15,23]. Distinct bands correspond to the monomeric and hexameric forms of the SatP. The molecular weight of monomeric SatP is 20.071 kDa, corresponding to the lowest band. Hexameric SatP was also detected, although the hexameric SatP migrated at a position lower than its theoretical molecular weight under denaturing conditions. This is consistent with migration patterns reported previously [15,23].

### 3.2. Single-Channel Recordings of SatP

**Figure 3:**
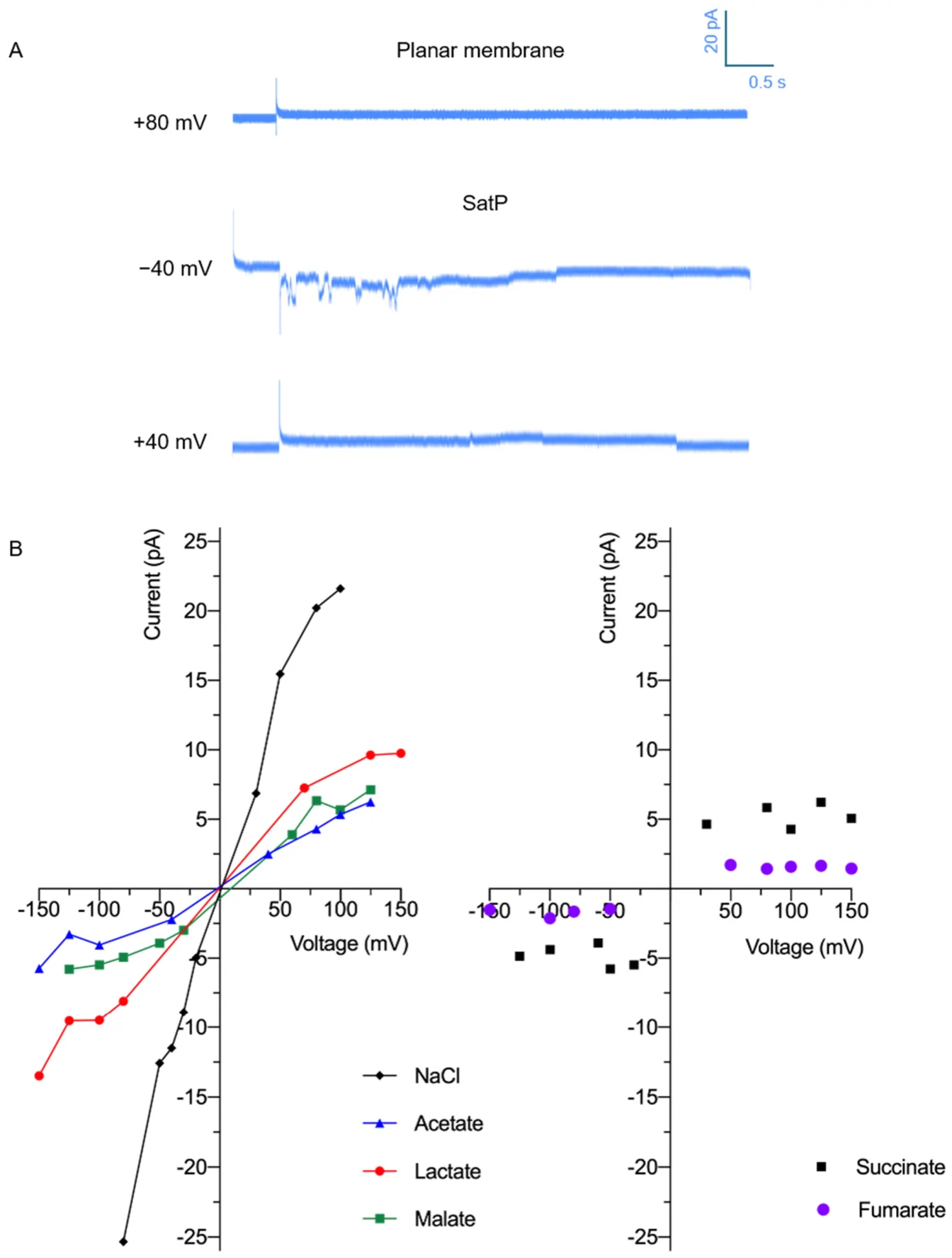
Single-channel recordings in the presence of various substrates. (**A**) Blank measurement in the absence of SatP at +80 mV in acetate buffer (upper trace), and representative single-channel recording traces at +40 mV and −40 mV in acetate buffer (lower traces). When a stepwise current change occurred only from level 0 to level 1, the step size was recorded. When more than one step occurred (e.g., from level 0 to level 1 and from level 1 to level 2), the size of each step was recorded, and the average step size was calculated. Current changes at a given voltage were recorded at least twice. (**B**) I–V curves of single-channel recordings using buffers comprising different substrates or NaCl as indicated in the plots. Linear I–V curves are presented on the left side (*p* ≤ 0.0001), and non-linear I–V curves are presented on the right.

Before measurements in the presence of various substrates were conducted, two different single-channel recordings were performed as the negative and positive control, respectively. First, blank measurements were conducted in acetate buffer without SatP, which showed no detectable current changes under varying applied voltages (Figure 3A). This result confirms the formation of intact, electrically insulating lipid bilayers. Subsequently, cell-free synthesized SatP in liposomes was added in NaCl buffer as a positive control. Application of different voltage steps resulted in a pronounced current response with a linear current–voltage relationship, indicating that the in vitro synthesized SatP was functionally incorporated into the planar lipid bilayer (Figure 3A).

Following these control experiments, cell-free synthesized SatP reconstituted in liposomes was introduced into the chamber in acetate buffer, and channel activity was examined. Distinct current changes were detected at different voltages (Figure 3A), demonstrating that liposome(s) fused with the planar DPhPC membrane and the reconstituted SatP retained functional activity in the presence of acetate. The corresponding current steps recorded at each voltage were plotted to generate an I–V curve, which exhibited a linear relationship (Figure 3B). Based on the slope of the I–V plot, the conductance of cell-free expressed SatP reconstituted in the membrane was determined to be 39.93 pS (Table 1).

Following the analysis in acetate buffer, two additional SatP substrates, lactate and succinate, were examined using the same single-channel recording approach. Lactate produced a linear I–V relationship (Figure 3B), yielding a conductance of 78.15 pS (Table 1). In contrast, recordings obtained in the presence of the dicarboxylic acid succinate exhibited a non-linear I–V relationship (Figure 3B), precluding reliable determination of the SatP conductance in the presence of succinate.

As succinate transporters have previously been reported to transport other C4 organic acid anions, including malate and fumarate [24,25], these additional dicarboxylates were also examined using single-channel recordings. Current changes were detected for both substrates. In the presence of malate, a linear I–V relationship (Figure 3B) was observed, yielding a SatP conductance of 57.80 pS (Table 1). In contrast, with fumarate, only low current amplitudes across the applied voltage range were generated, resulting in a non-linear I–V relationship (Figure 3B) that precluded reliable determination of the SatP conductance in the presence of fumarate. Nevertheless, the measurable current responses observed for both malate and fumarate indicate that SatP also transports these C4 dicarboxylates. Overall, the tested substrates exhibited distinct I–V characteristics, indicating substrate-specific interactions with SatP that reflect differences in their physicochemical properties, which are further discussed below.

### 3.3. An Initial Strategy for Acetate Transporter Screening

**Figure 4:**
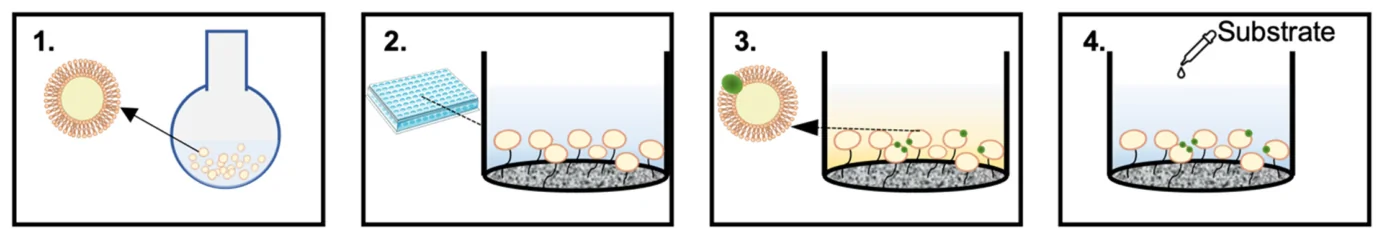
Plate-based screening assay. (**1**). Unilamellar liposome production. (**2**). Biotin-labeled liposomes produced in the first step were immobilized into wells coated with streptavidin. (**3**). Cell-free expression mix is added into wells for the expression of target transporters. (**4**). After the expression and reconstitution of the transporter proteins, substrate is added from the outside of proteoliposomes and the transport can be measured, e.g., by encapsulating fluorescent indicators inside the liposomes to detect substrate uptake in a fluorescence-based assay.

**Figure 5:**
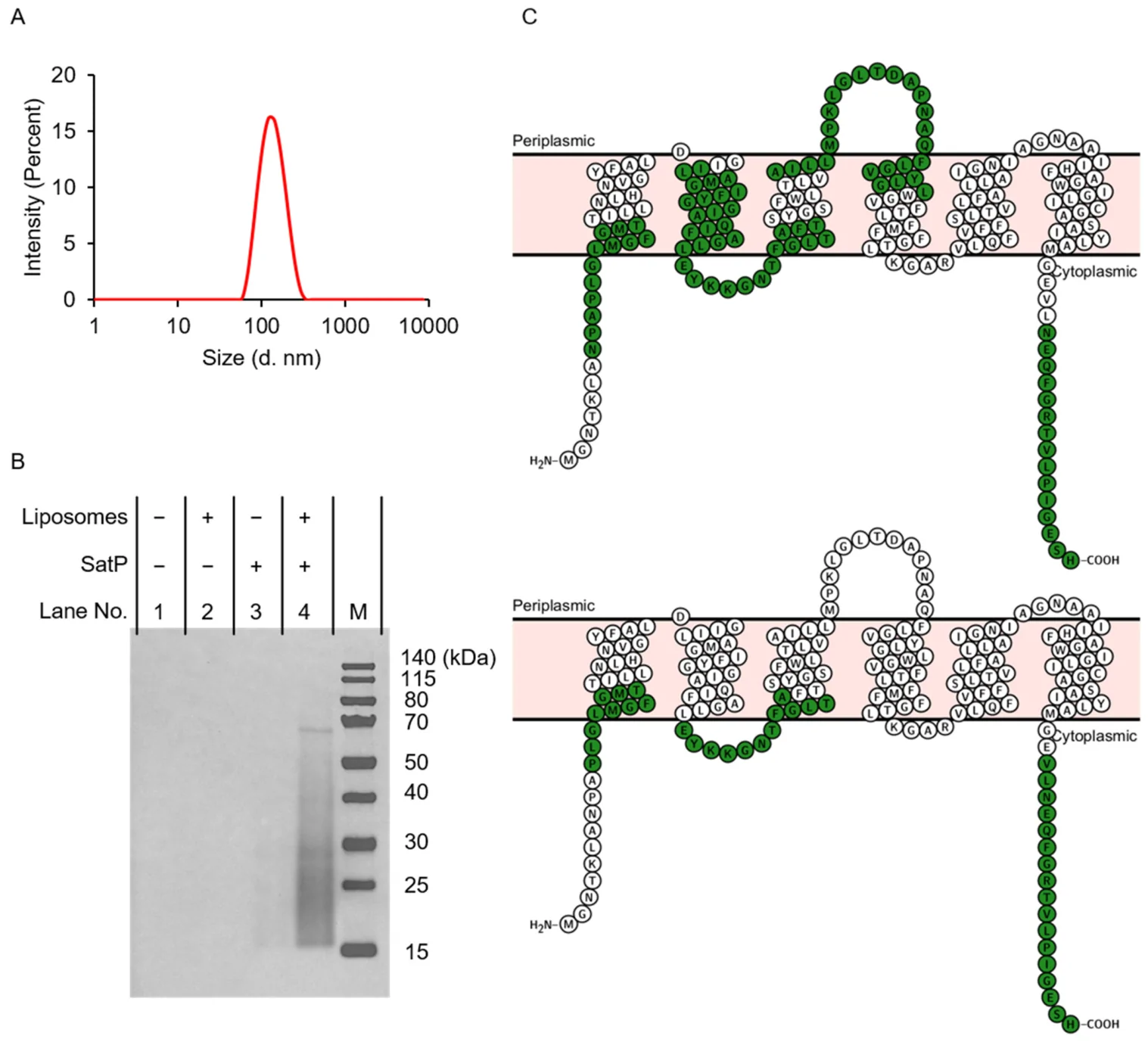
Characterization of the screening assay. (**A**) Representative size distribution profile of liposomes. (**B**) Western blot analysis of SDS-PAGE. The solubilized fractions from wells expressed in different conditions—with or without liposomes, and with or without SatP-encoding plasmids. (**C**) Peptide-coverage maps from two independent plate wells. Peptides identified by mass spectrometry are highlighted in green; membrane bilayer is indicated in light pink, with the periplasmic side at the top and the cytoplasmic side at the bottom.

Following confirmation of functional SatP expression in the cell-free system, a preliminary screening assay was designed to enable the characterization of acetate transporters. The assay was implemented in a microtiter plate (Figure 4). First, unilamellar and biotin-labeled liposomes were prepared, exhibiting an average diameter of 170 nm and a polydispersity index (PDI) of 0.232 (Figure 5A). Subsequently, the liposomes were incubated in streptavidin-coated wells to enable immobilization on the plate surface. The cell-free expression mixture was then added to initiate the SatP expression and reconstitution on the immobilized liposomes (Figure 4). To evaluate assay feasibility, control conditions lacking either immobilized liposomes or SatP-encoding plasmid were included. SatP was detected in solubilized fractions by Western blot analysis exclusively in samples containing both liposomes and plasmid DNA (Figure 5B).

To investigate the topology of SatP insertion into the liposomes, protease digestion was performed following the cell-free expression. Two wells were treated with proteases to selectively digest peptide regions exposed on the exterior side of the liposomal bilayer. Subsequent mass spectrometry analysis showed that the majority of detected peptides did not correspond to predicted transmembrane regions of SatP (Figure 5C). This observation confirmed the insertion of SatP into the liposomal membrane rather than non-specific association with the liposome surface, as surface-associated proteins would predominantly yield peptides derived from transmembrane regions.

The identified peptides originated from both cytoplasmic and periplasmic domains, indicating bidirectional insertion of SatP into the liposomal membrane. Nevertheless, a preferential orientation with the cytoplasmic side facing outward from the liposomes was observed. Although this orientation is opposite to that in *E. coli* cells, where the cytoplasmic side faces the cell interior, it is consistent with the experimental setup, in which the cell-free expression mixture was added to the external environment of the liposomes, resulting in protein synthesis occurring from the outside of the liposomes and thus in the cytoplasmic side facing the outside of the liposomes. Although adopting an orientation opposite to its native membrane orientation, it has been reported that SatP mediates bidirectional transport [15]. Therefore, the insertion orientation does not substantially influence transport activity.

## 4. Discussion

### 4.1. Cell-Free Expression of SatP

This study demonstrated the functional production of SatP using a cell-free expression system in liposomes. To the best of our knowledge, this represents the first detailed report of functional SatP expression achieved through cell-free synthesis. Considering the inherent challenges associated with membrane protein expression, particularly for multi-pass transmembrane proteins such as SatP, these findings constitute an important advance in the in vitro expression and characterization of membrane transporters.

In the cell-free expression of this study, SatP was expressed in the presence of not only liposomes but also the detergent DDM. However, SatP produced in the presence of DDM exhibited no detectable acetate transport activity. This observation highlights the critical importance of the lipid environment for the nascent SatP to adopt its functional structure. Liposomes provide a bilayer lipid environment that closely resembles the native biological membrane, whereas DDM solubilizes membrane proteins in the form of detergent micelles, which lack a bilayer structure [26]. Previous studies have similarly emphasized the essential role of lipid environments in membrane protein functionality [27,28]. The present findings further support the importance of an appropriate membrane environment for SatP, as demonstrated by the difference in transport activity observed between the different membrane mimetics.

Another possible explanation for the lack of activity in DDM micelles is that the conformational states required for substrate transport may not be populated under these conditions. A conformational-related transport mechanism was proposed by Wu et al. [29], based on molecular dynamics simulations of SatP from *C. koseri*. The key to transport comes from a few amino acids at the narrowest site of the channel of monomeric SatP, the so-called constriction site in their study. The different positioning of these key amino acids around the constriction site results in either open or closed states of the transport channel. In the open state, SatP-mediated transport occurs, while in the closed state, SatP is not transported.

Given the structural similarity between *E. coli* and *C. koseri* SatP and the conservation of the amino acid residues forming the constriction site, both succinate–acetate transporters are likely to share a similar transport mechanism [15,16].

### 4.2. Substrate–SatP Interplay

Distinct current–voltage characteristics were observed among the tested substrates. Acetate, lactate, and malate exhibited linear I–V relationships (Figure 3B), indicating that the measured currents increased proportionally with the applied voltage. In contrast, succinate and fumarate displayed non-linear I–V relationships, suggesting a different interaction with SatP. These differences may arise from the physicochemical properties of the substrates. Succinate and fumarate are symmetric dicarboxylates carrying two negative charges and lacking additional functional groups, which may promote stronger and more uniform electrostatic interactions with the channel. Such interactions could increase substrate residence time within the channel of monomeric SatP, causing current amplitudes to be influenced by substrate binding and release rather than scaling linearly with the applied voltage.

In contrast, acetate and lactate are monocarboxylates carrying a single negative charge and are therefore expected to interact weakly with charged residues lining the channel. Consequently, their passage through the channel is more strongly influenced by the applied voltage, resulting in linear I–V relationships. Although malate also carries two negative charges, the presence of a hydroxyl group introduces molecular asymmetry and additional hydrogen-bonding potential. These features may alter its interaction with the channel and account for its linear I–V behavior despite its dicarboxylate structure.

### 4.3. Development of an In Vitro Screening Assay

With its open and flexible production format, cell-free expression has emerged as a versatile platform for rapid protein production and characterization, offering significant advantages for in vitro protein screening applications. In this study, we explored a strategy toward establishing an in vitro platform for carbohydrate transporter screening. The experimental setup was inspired by a fluorescence-based approach previously developed for measuring weak acid permeation across membranes [30]. Through the initial characterization of the plate-based assay using SatP, we confirmed that this strategy enables the successful incorporation of SatP into liposomes. By encapsulating labeled substrates within the liposome lumen during preparation, it would be possible to directly assess transporter activity in future studies.

Due to the inherent difficulties associated with membrane protein expression, considerable efforts have been made to establish cell-free systems for the high-throughput screening of membrane protein expression, and protocols combining different membrane-mimetic strategies have been developed [7,8]. In contrast, functional screening remains much more challenging because membrane proteins exhibit diverse functions and often require protein-specific functional assays [8,9,10].

Some functional assays have included the use of radiolabeled substrates [8] and cell-free expression combined with electrophysiological measurements [9], the latter being conceptually similar to the single-channel recordings presented here. Fujii et al. established a functional screening platform for α-hemolysin and its mutants [10]. However, the assay required access to fluorescence-activated cell sorting (FACS) instrumentation and the availability of fluorescently labeled substrates.

In the strategy presented here, we designed an assay that is more readily accessible. Owing to the symport function of SatP [11], acetate can be transported together with H^+^; therefore, transport activity can be monitored by encapsulating pH indicators inside the liposomes. This approach has the potential to be extended to other transporters, particularly symporters that couple substrate transport with H^+^ translocation. The broad applicability of this strategy therefore offers considerable potential for the screening of acetate transporters and, more generally, other weak acid transporters.

While the screening assay requires further validation and optimization, the initial characterization presented here demonstrates a convenient and rapid strategy for transporter screening. Such a screening platform could facilitate the identification and selection of transporters capable of enhancing microbial growth under different fermentative conditions, thereby contributing to improved biofuel production processes.

## 5. Conclusions

This study highlights the potential of cell-free membrane protein synthesis as a powerful approach for investigating transporter function in a controlled and customizable environment. The results demonstrate that the functional state of SatP is strongly influenced by its surrounding membrane environment, underscoring the importance of lipid–protein interactions in transporter activity. Furthermore, the observed substrate-specific transport characteristics suggest that subtle differences in molecular structure can markedly affect permeation behavior and channel interactions. Beyond providing new insights into SatP function, the combination of cell-free expression and liposome-based reconstitution establishes a versatile experimental framework for mechanistic studies and transporter discovery. Such platforms may accelerate the identification and engineering of transport systems for biotechnological applications, particularly in the optimization of microbial production processes.

## Figures and Tables

**Table 1:** The conductance of SatP measured by single-channel recordings.

Substrate	Conductance	Linear Fit Equation	R*^2^*
NaCl	272.1 pS	Y = 0.272 × X − 0.71	0.95
Acetate	42.29 pS	Y = 0.042 × X + 0.85	0.91
Lactate	77.69 pS	Y = 0.078 × X − 0.99	0.92
Malate	56.79 pS	Y = 0.057 × X + 0.17	0.96
Fumarate	Non-linear		
Succinate	Non-linear		

## Data Availability

The data presented in this study are available on request from the corresponding authors.

## Appendix A

**Table A1:** Buffer conditions used in the single-channel recordings.

Tested Molecule	Recipe				
NaCl	200 mM	NaCl	10 mM	HEPES	pH 7.0
Acetate	200 mM	acetate	10 mM	HEPES	pH 7.0
Lactate	200 mM	lactate	10 mM	HEPES	pH 7.0
Malate	200 mM	malate	10 mM	HEPES	pH 7.0
Fumarate	200 mM	fumarate	10 mM	HEPES	pH 7.0
Succinate	200 mM	succinate	10 mM	HEPES	pH 7.0
